# Extremelt low rates of hypoglycemia following transfer of tight glucose control management to ICU nurses

**DOI:** 10.1186/1753-6561-5-S6-P35

**Published:** 2011-06-29

**Authors:** C Joseph, F Delodder, T Szostek, P Maravic, M-D Schaller, M Berger, J-P Revelly, P Eggimann, the staff of the Service de Médecine Intensive Adulte (SMIA)

**Affiliations:** 1 Intensive Care Medicine, CHUV, Lausanne, Switzerland

## Introduction / objectives

Tight glucose control (TGC) failed to improve survival and has been associated with high rates (6-18%) of hypoglycemia (< 2.5mmol/l), themselves associated with significant mortality. Blood glucose levels vary largely in critically ills and TCG requires individualized knowledge of the patient condition. Bedside frequent blood glucose measurements are mandatory for continue adaptations of insulin and glucose administration. We progressively transfer TGC to the nursing personal.

## Methods

TGC was introduced in 2003 and then progressively transferred from physicians to nurses since 2007. Nurses are specifically trained to adapt infusion rates of nutrition and insulin according to medically predefined targets (4.5–6.0; 6.0–8.0; >10mmol/l). Glucose levels are determined by the central lab. or by ICU blood gas analyzers. Glycemia (n=809’725) were extracted from our electronic clinical information system (Metavision®) and analyzed with STATA.

## Results

A continuous decrease in yearly standard deviations (IQR), suggest that TGC was effectively improved (Table).

We suppressed the lowest target (4.5–6.0 mmol/l) in May 2009 and this may explain the mean increase since then. The rate of hyperglycemia decreases below 10%, with rates of hypoglycemia (<2.5 mmol/l) largely below 0.4%, 50 to 100 fold lower than those reported in the literature.

## Conclusion

Implementation and transfer of tight glucose control in a large mixed adult ICU significantly decreases the rate of hyperglycemia to less than 10%, with extremely low rates of hypoglycemia (< 0.1%). These results strongly suggest that transfer of tight glucose control to bedside nursing personal is safe and effective.

## Disclosure of interest

None declared.

